# *Lhx8* regulates primordial follicle activation and postnatal folliculogenesis

**DOI:** 10.1186/s12915-015-0151-3

**Published:** 2015-06-16

**Authors:** Yu Ren, Hitomi Suzuki, Krishna Jagarlamudi, Kayla Golnoski, Megan McGuire, Rita Lopes, Vassilis Pachnis, Aleksandar Rajkovic

**Affiliations:** Magee-Womens Research Institute, Department of Obstetrics, Gynecology and Reproductive Sciences, University of Pittsburgh, Pittsburgh, PA 15213 USA; Department of Pathology, University of Pittsburgh, Pittsburgh, PA 15213 USA; Department of Human Genetics, University of Pittsburgh, Pittsburgh, PA 15213 USA; Department of Experimental Animal Model for Human Disease, Graduate School of Medical and Dental Sciences, Tokyo Medical and Dental University, Bunkyo-ku, Tokyo 113-8510 Japan; Division of Molecular Neurobiology, MRC National Institute of Medical Research, London, NW7 1AA UK

**Keywords:** Lhx8, Primordial follicle, Oocyte, Lin28a, Ovarian reserve

## Abstract

**Background:**

The early stages of ovarian follicle formation—beginning with the breakdown of germ cell cysts and continuing with the formation of primordial follicles and transition to primary and secondary follicles—are critical in determining reproductive life span and fertility. Previously, we discovered that global knockouts of germ cell-specific transcriptional co-regulators *Sohlh1*, *Sohlh2*, *Lhx8*, and *Nobox*, cause rapid oocyte loss and ovarian failure. Also factors such as *Nobox* and *Sohlh1* are associated with human premature ovarian failure. In this study, we developed a conditional knockout of *Lhx8* to study oocyte-specific pathways in postnatal folliculogenesis.

**Results:**

The conditional deficiency of *Lhx8* in the oocytes of primordial follicles leads to massive primordial oocyte activation, in part, by indirectly interacting with the PI3K-AKT pathway, as shown by synergistic effects on FOXO3 nucleocytoplasmic translocation and rpS6 activation. However, LHX8 does not directly regulate members of the PI3K-AKT pathway; instead, we show that LHX8 represses *Lin28a* expression, a known regulator of mammalian metabolism and of the AKT/mTOR pathway. LHX8 can bind to the *Lin28a* promoter, and the depletion of *Lin28a* in *Lhx8*-deficient oocytes partially suppresses primordial oocyte activation. Moreover, unlike the PI3K-AKT pathway, LHX8 is critical beyond primordial follicle activation, and blocks the primary to secondary follicle transition.

**Conclusions:**

Our results indicate that the LHX8-LIN28A pathway is essential in the earliest stages of primordial follicle activation, and LHX8 is an important oocyte-specific transcription factor in the ovary for regulating postnatal folliculogenesis.

**Electronic supplementary material:**

The online version of this article (doi:10.1186/s12915-015-0151-3) contains supplementary material, which is available to authorized users.

## Background

Following the proliferation of primordial germ cells, oogonia enter meiosis circa embryonic day 13 (E13.5) and are then referred to as oocytes in the embryonic mouse ovary. Oocytes in the embryonic ovary exist in clusters, surrounded by epithelial pre-granulosa cells [1]. Germ cell cyst breakdown results in the envelopment of individual oocytes by flat granulosa cells to form primordial follicles. Primordial follicles contain small oocytes (<20 μm) surrounded by flat granulosa cells. These early steps in the formation of primordial follicles are critical, since primordial follicles constitute the fundamental reproductive units of the ovary and give rise to all dominant follicles. Primordial follicle activation (PFA) is a process by which primordial follicles are selected into the growing follicle pool [2]. Morphologically, PFA is characterized by oocyte growth to greater than 20 μm and transition of the flat epithelial layer to cuboidal granulosa cells. PFA is independent of gonadotropins [3], occurs prior to puberty, and is spontaneously observed when an ovary is transplanted or cultured in vitro [4]. Several groups have shown the importance of the ubiquitous PI3K-AKT-mTORC1 pathways within oocytes in regulating PFA [5–9]. Conditional ablation of *Foxo3*, *Pten*, and *Tsc1*/*2* in oocytes triggers massive oocyte activation [5, 6, 8, 10]. Activated oocytes in these animals survive beyond 5 weeks, perhaps not surprising given the importance of this pathway in regulating apoptosis and cell death.

The role of oocyte-specific pathways in oocyte activation is unknown. *Lhx8* encodes a highly conserved LIM homeodomain protein that is preferentially expressed in mammalian ovaries, including human ovaries [11, 12]. Female mice with global *Lhx8* deficiency are infertile and rapidly lose oocytes after birth [13]; so great is the loss in *Lhx8* global knockout that few oocytes remain by postnatal day 7. To study the postnatal role of *Lhx8* in oocyte activation, we conditionally ablated *Lhx8* in oocytes of primordial follicles (primordial oocytes). Our results indicate that *Lhx8* represses oocyte activation and plays a dominant role over PTEN-led pathways in oocyte survival. *Lhx8* depletion in primordial oocytes decouples oocyte activation from somatic differentiation. Moreover, *Lhx8* directly regulates *Lin28a* expression and indirectly interacts with the PI3K-Akt pathway to effect repression of PFA. Furthermore, unlike the *Pten* pathway, we found that conditional deletion of *Lhx8* from oocytes of primary follicles (primary oocytes) causes primary follicle death and depletion of the secondary/antral follicle pool.

## Results

### Conditional depletion of *Lhx8* by *Gdf9Cre* causes massive primordial follicle activation

We previously reported that global knockout of *Lhx8* causes infertility and loss of oocytes by postnatal day 7 (PD7) [14]. In the global knockout of *Lhx8*, primordial-like follicles form (oocytes less than 20 μm in diameter and surrounded by flat granulosa cells), but oocytes do not grow. Since *Lhx8* is expressed in both embryonic and postnatal female germ cells, it is possible that global knockout of *Lhx8* disrupts early embryonic pathways that lead to postnatal oocyte depletion. We therefore investigated the postnatal functions of *Lhx8* by generating a conditional knockout mouse, using a floxed *Lhx8* allele (*Lhx8*^*flx*/*flx*^) [15] and a *Gdf9Cre* transgenic mouse [16]. The *Gdf9Cre* transgene will inactivate *Lhx8* specifically in primordial oocytes. *Gdf9Cre* is highly efficient in oocytes and, when present in either *Lhx8*^*flx*/*flx*^ or *Lhx8*^*flx*/-^ animals, displayed the same ovarian phenotype. We used *Lhx8*^*flx*/*flx*^*Gdf9Cre* to study the effects of *Lhx8* conditional deficiency in primordial follicles on ovarian development.

**Fig. 1:**
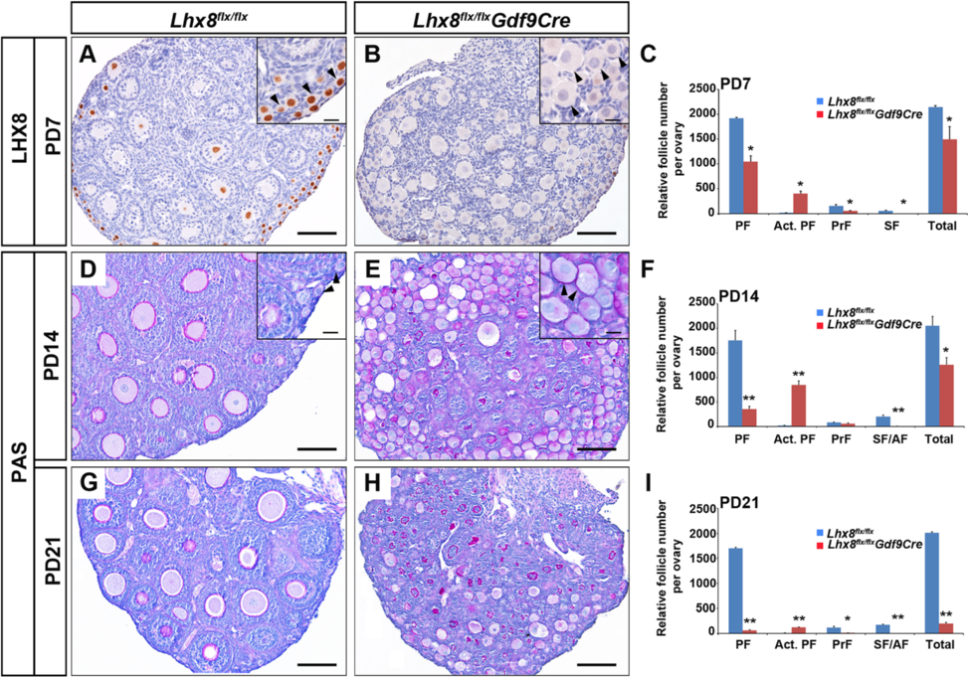
Postnatal inactivation of *Lhx8* causes premature activation of primordial follicles and ovarian failure. **a** and **b** Anti-LHX8 antibodies were used to detect oocytes in paraformaldehyde-fixed and hematoxylin-counterstained ovaries taken at postnatal day 7 (PD7). Ovaries were derived from control (*Lhx8* ^*flx*/*flx*^, A) and *Lhx8* conditional knockout (*Lhx8* ^*flx*/*flx*^ *Gdf9Cre*, B) mice. *Arrowheads* in the *inset* of panel A indicate primordial follicles that stain with anti-LHX8 antibodies (brown signal). *Arrowheads* in the *inset* in panel B show activated primordial follicles (oocytes larger than 20 μm without cuboidal granulosa cells). **d**, **e**, **g** and **h** Periodic acid–Schiff (PAS) staining of PD14 (D and E) and PD21 (G and H) ovaries derived from control (D and G) and *Lhx8* conditional knockout (E and H) mice. *Arrowheads* in the *inset* of panels D and E indicate primordial activated primordial follicles. **c**, **f** and **i** Quantification of ovarian follicle types in *Lhx8* ^*flx*/*flx*^ and *Lhx8* ^*flx*/*flx*^ *Gdf9Cre* mice. Five pairs of ovaries from PD7 (C), PD14 (F), and PD21 (I) were embedded in paraffin and serially sectioned at 5 μm thickness, and the follicles were counted. Anti-NOBOX antibodies stain oocyte nuclei throughout folliculogenesis and were used to identify oocytes in our counts. Every fifth section was counted. We scored primordial follicles (PF), activated primordial follicles (Act. PF, oocyte diameter greater than 20 μm without cuboidal granulosa cells), primary follicles (PrF) and secondary/antral follicles (SF/AF). *P < 0.05, **P < 0.01. Scale bars: 100 μm (A, B, D, E, G and H); 20 μm (*inset* in A, B, D and E)

At PD7 and PD14, LHX8 protein was depleted in *Lhx8*^*flx*/*flx*^*Gdf9Cre* ovaries and massive oocyte activation occurred in the primordial follicles, as manifested by oocytes reaching a diameter greater than 20 μm without significant transformation of the surrounding flat granulosa cells (Fig. 1a–f). At PD7, *Lhx8*^*flx*/*flx*^*Gdf9Cre* mice had 398 ± 53 activated primordial follicles per ovary compared to 16 ± 2 per ovary in *Lhx8*^*flx*/*flx*^ controls. The number of primordial follicles was 1917 ± 23 per ovary in *Lhx8*^*flx*/*flx*^ controls and was significantly reduced to 1043 ± 119 per ovary in *Lhx8*^*flx*/*flx*^*Gdf9Cre* mice (Fig. 1c). We detected a decline in primary follicles in *Lhx8*^*flx*/*flx*^*Gdf9Cre* mice, implying a block in the transition from activated primordial follicles to primary follicles. There were a negligible number of advanced follicle types (oocytes surrounded by multiple layers of granulosa cells) in PD7 *Lhx8*^*flx*/*flx*^*Gdf9Cre* ovaries, compared to the *Lhx8*^*flx*/*flx*^ controls.

At PD14, *Lhx8*^*flx*/*flx*^*Gdf9Cre* ovaries contained 847 ± 82 activated primordial follicles per ovary, compared to 15 ± 7 per ovary in *Lhx8*^*flx*/*flx*^ controls. The number of primordial follicles significantly diminished from 1753 ± 204 in *Lhx8*^*flx*/*flx*^ controls to 353 ± 58 in *Lhx8*^*flx*/*flx*^*Gdf9Cre* ovaries (Fig. 1f). By PD21, the total number of follicles from primordial to secondary was greatly diminished in the *Lhx8*^*flx*/*flx*^*Gdf9Cre* ovary (Fig. 1g–i). By PD35, there were barely any oocytes and follicles detected in the *Lhx8*^*flx*/*flx*^*Gdf9Cre* ovary (see Additional file 1: Figure S1C) and *Lhx8*^*flx*/*flx*^*Gdf9Cre* females were sterile (see Additional file 2: Figure S2A). Our studies show that postnatal inactivation of *Lhx8* within oocytes of primordial follicles leads to massive oocyte activation, decoupling of oocyte activation from somatic cell transformation, oocyte death, and infertility.

### LHX8 interacts with PI3K-AKT pathway

**Fig. 2:**
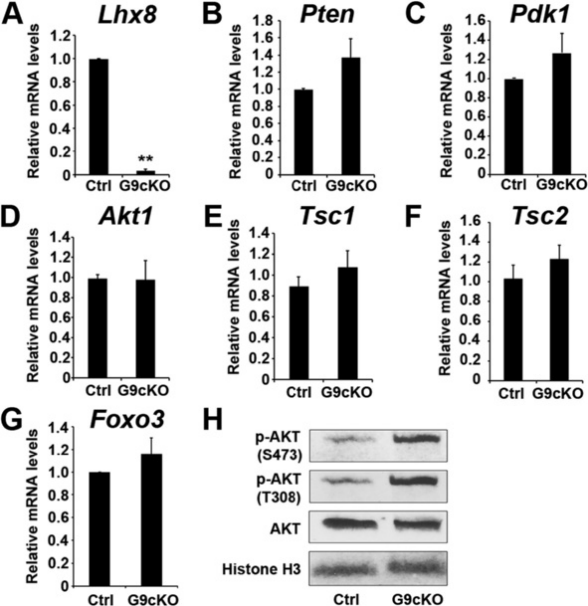
AKT is activated in *Lhx8* ^*flx*/*flx*^ *Gdf9Cre* oocytes. **a**–**g** Oocytes were isolated from PD7 control (*Lhx8* ^*flx*/*flx*^, Ctrl) and *Lhx8* ^*flx*/*flx*^ *Gdf9Cre* (G9cKO) mouse ovaries and RNA was extracted for cDNA conversion and real-time quantitative polymerase chain reaction (RT-qPCR). Data were normalized to *Gapdh* expression and are given as the mean relative quantity (compared with control), with error bars representing the standard error of the mean. Student’s *t*-test was used to calculate *P* values. The only significant difference was noted in the expression of *Lhx8*, as expected. ** *P* < 0.01. **h** Oocytes were isolated from PD7 control and *Lhx8* ^*flx*/*flx*^ *Gdf9Cre* ovaries, protein was extracted, and a Western blot test was performed on three independent samples, using antibodies against AKT and its two phosphorylated forms (S473 and T308). Histone H3 immunoreactivity was used as a control

We examined RNA expression of genes that encode important members of the PI3K-AKT-mTOR pathways in PD7 *Lhx8*^*flx*/*flx*^*Gdf9Cre* oocytes. Real-time polymerase chain reaction (PCR) did not detect significant changes in the expression of *Pten*, *Akt*, *Foxo3*, *Pdk1*, *Mtor*, *Rps6*, *Deptor*, *Rictor*, or *Tsc1*/*2* (some of these are shown in Fig. 2). These results indicate that LHX8 does not directly affect transcription of a subset of genes known to encode proteins in the PI3K-AKT-mTOR pathways. Then we assessed whether the PI3K-AKT pathway was activated at the protein level in *Lhx8*^*flx*/*flx*^*Gdf9Cre* oocytes. AKT is a serine/threonine-specific protein kinase that plays a key role in apoptosis and PFA. Phosphorylated AKT activates multiple downstream pathways, including FOXO3 phosphorylation, resulting in oocyte activation [8]. Western blot analyses on oocytes from PD7 ovaries showed that phosphorylation of AKT at two sites, S473 and T308, was higher in *Lhx8*^*flx*/*flx*^*Gdf9Cre* oocytes than in controls (Fig. 2h). But interestingly, only p-AKT (T308) was detected in the activated primordial follicles (see Additional file 3: Figure S3).

**Fig. 3:**
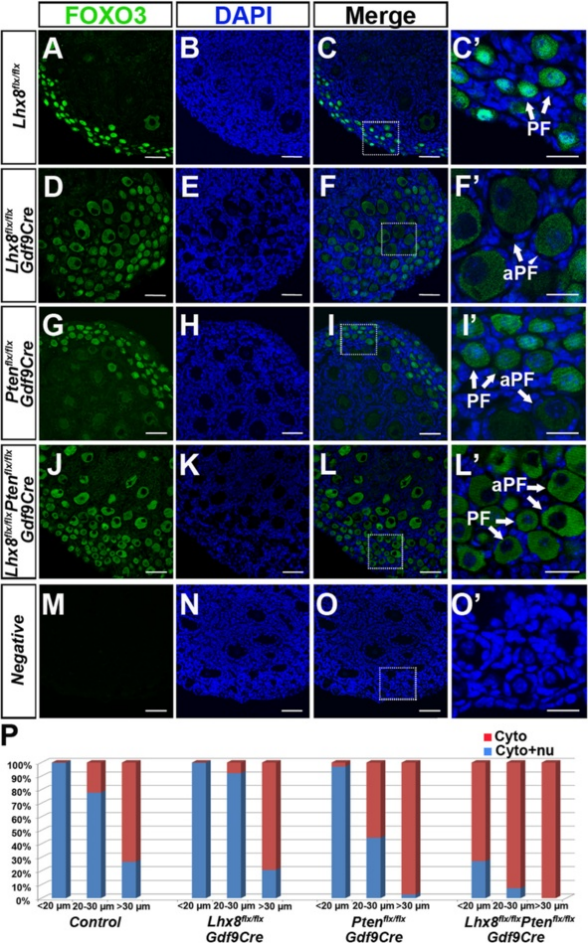
*Lhx8 and Pten* conditional knockout effects on FOXO3 localization. **a**–**c** In control mice (*Lhx8* ^*flx*/*flx*^), FOXO3 is expressed in the nucleus and cytoplasm of primordial oocytes (PF, *arrows* in **c**'). **d**–**f** In *Lhx8* ^*flx*/*flx*^ *Gdf9Cre* mice, the extensive nucleocytoplasmic translocation is not observed in activated primordial follicles between 20 and 30 μm (aPF, *arrow* in **f**') but is noted in activated primordial follicles larger than 30 μm (aPF, *arrow* in F'). **d**–**i** A similar expression pattern of FOXO3 localization exists in *Pten* conditional knockout (*Pten* ^*flx*/*flx*^ *Gdf9Cre*) mice. The *arrows* in **i**' represent primordial follicles (PF) with both nuclear and cytoplasm expression of FOXO3 and cytoplasm expression of FOXO3 in activated primordial follicles (aPF) below 30 μm. **j**–**l** However, in mice that are conditionally deficient in both *Lhx8* and *Pten* (*Lhx8* ^*flx*/*flx*^ *Pten* ^*flx*/*flx*^ *Gdf9Cre*), FOXO3 nucleocytoplasmic translocation is present in primordial, activated, and primary oocytes. The negative control is immunofluorescence in the presence of secondary antibodies and is shown in **m**–**o**. The *boxed areas* in C, F, I, L, and O are shown magnified in C', F', I', L', and O'. **p** Graphic representation of FOXO3 distribution (cytoplasm only or nucleus and cytoplasm). Oocytes were grouped by size (diameter) as less than 20 μm, between 20 and 30 μm, and greater than 30 μm. Only oocytes with clear DAPI nuclear staining were counted. Scale bars: 50 μm (A–C, D–F, G–I, J–L, and M–O); 20 μm (C', F', I', L', and O')

Previous studies have shown FOXO3 nucleocytoplasmic translocation and rpS6 phosphorylation via the PI3K-AKT-mTOR pathways to be associated with PFA [5, 6, 8]. We examined FOXO3 nucleocytoplasmic translocation and rpS6 phosphorylation in PD7 *Pten* and *Lhx8* conditional knockouts (Fig. 3 and Additional file 4: Figure S4). FOXO3 nucleocytoplasmic translocation was prominent in oocytes larger than 30 μm but did not show obvious translocation among oocytes between 20 and 30 μm in *Lhx8*^*flx*/*flx*^*Gdf9Cre* mice (Fig. 3d–f, f', p). Oocytes of primordial follicles in *Pten* and *Lhx8* conditional single knockouts as well as corresponding controls did not show FOXO3 nucleocytoplasmic translocation. However, FOXO3 nucleocytoplasmic translocation was significantly induced in the primordial (<20 μm) oocytes (Fig. 3j–l, l', p) of the PD7 double *Lhx8*/*Pten* conditional knockouts (*Lhx8*^*flx*/*flx*^*Pten*^*flx*/*flx*^*Gdf9Cre*). These data indicate a synergistic action of the LHX8 and PTEN proteins on FOXO3 nucleocytoplasmic translocation.

We also examined the effects of double *Pten* and *Lhx8* deficiency on rpS6. mTORC1 promotes protein translation and cell growth, in part, through activation of S6K1 (by phosphorylation of its threonine 389) and through phosphorylation and inactivation of eIF4E-binding proteins. S6K1 is responsible for phosphorylation and activation of rpS6, which leads to enhanced protein translation and ribosome biogenesis. In control, *Lhx8*^*flx*/*flx*^*Gdf9Cre*, and *Pten*^*flx*/*flx*^*Gdf9Cre* ovaries, rpS6 was only significantly activated in growing follicles at PD7 (see Additional file 4: Figure S4). However, the PD7 *Lhx8*^*flx*/*flx*^*Pten*^*flx*/*flx*^*Gdf9Cre* ovaries showed significant activation of rpS6 in primordial (<20 μm) oocytes (see Additional file 4: Figure S4D, D'). These results further indicate that the *Lhx8* and *Pten* pathways synergistically interact to accelerate two events associated with PFA—FOXO3 nucleocytoplasmic translocation and phosphorylation of rpS6.

### *Lin28a* RNA and protein expression are upregulated in Lhx8flx/flxGdf9Cre oocytes

LHX8 is a transcription factor, and we expect that its major effect will be at the RNA level. We therefore analyzed the transcriptome of *Lhx8*^*flx*/*flx*^*Gdf9Cre* ovaries via high-throughput RNA sequencing (RNA-seq). We sequenced 180 million tags in each sample and compared the relative abundance of RNA tags encoded by genes in the PI3K-AKT-mTOR pathways derived from PD7 *Lhx8*^*flx*/*flx*^*Gdf9Cre* and control ovaries. No significant differences in RNA expression of PI3K-AKT-mTOR pathway genes (*Pik3r1*, *Pik3ca*, *Kit*, *Kitl*, *Gsk3b*, *Cdnd1*, *Wee1*, *Cdkn1a*/*b*, *Rheb*, *Mlst8*, *Mapkap1*, *Prr5*, and *Eif4ebp1*) were evident in the RNA-seq experiment (see Additional file 5: Table S1).

**Fig. 4:**
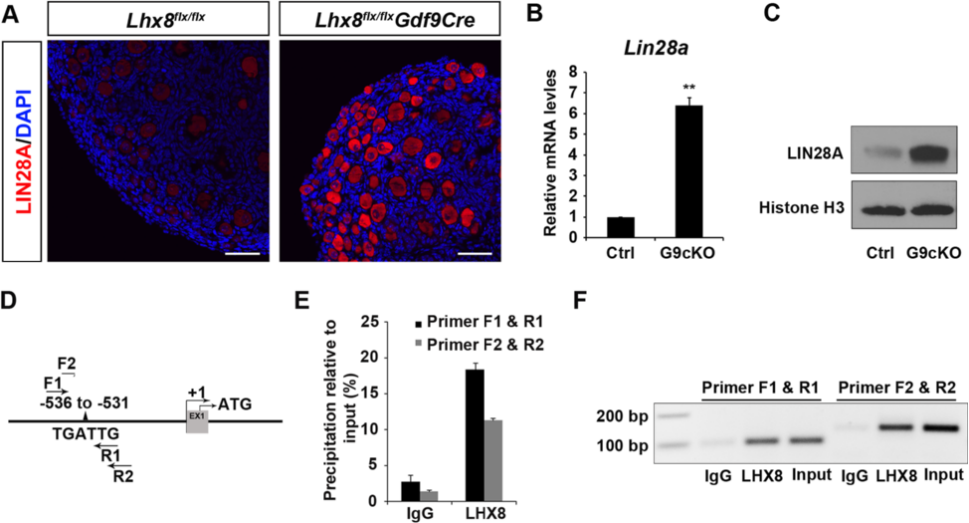
*Lhx8* suppresses *Lin28a* expression. **a** Immunofluorescence with anti-LIN28A antibodies shows that LIN28A is preferentially expressed in oocytes within the ovary. LIN28A abundance is higher in *Lhx8* conditionally deficient oocytes (*Lhx8* ^*flx*/*flx*^ *Gdf9Cre*) compared to controls (*Lhx8* ^*flx*/*flx*^). **b** and **c** *Lin28a* transcripts and protein are significantly more highly expressed in *Lhx8* ^*flx*/*flx*^ *Gdf9Cre* oocytes (G9cKO) unlike controls (Ctrl). **a**–**f** Chromatin immunoprecipitation (ChIP) assays with anti-LHX8 affinity purified antibodies on oocytes. **d** A putative LHX8 DNA binding site, TGATTG [22], which perfectly fits the LHX8 binding consensus sequence, was identified at position −536 to −531 relative to the *Lin28a* transcription initiation site. **e** Anti-LHX8 antibodies precipitate genomic DNA containing the TGATTG binding sequence from the *Lin28a* promoter region as shown by ChIP-quantitative PCR (qPCR). Immunoglobulin G (IgG) antibodies served as control. The percentage input method is used to analyze the qPCR data. “Input” is the PCR product from chromatin pellets before immunoprecipitation. A triplicate average *Ct* normalized to an adjusted input was used for the calculation of percentage input. Two sets of primers (F1 and R1) and (F2 and R2) were used to perform ChIP-qPCR. **f** PCR amplification of the oocyte input DNA, as well as DNA precipitated by normal guinea pig IgG and anti-LHX8 antibodies by the F1/R1 and F2/R2 primer sets. Scale bars: 50 μm (A)

In addition to analyzing the expression of known PI3K-AKT-mTOR genes, we studied global differences in the transcriptome of *Lhx8*^*flx*/*flx*^*Gdf9Cre* and control ovaries. We detected a sixfold increase in *Lin28a* RNA transcripts in *Lhx8*^*flx*/*flx*^*Gdf9Cre* compared to control ovaries (Fig. 4b and Additional file 5: Table S1). Immunofluorescence and Western blot analysis with anti-LIN28A antibodies showed that LIN28A protein was expressed at a significantly higher level in *Lhx8*^*flx*/*flx*^*Gdf9Cre* oocytes compared to the controls (Fig. 4a, c).

LIN28A is an RNA-binding protein that blocks biogenesis of *let*-*7* microRNAs and a known regulator of mammalian body size and metabolism, including onset of menarche [17, 18]. *Lin28a* is preferentially expressed in oocytes and embryo stem cells [19, 20]. Moreover, the PI3K-AKT-mTOR pathways can be activated by LIN28A [21]. LIN28A, therefore, may play a role in oocyte activation and growth.

### LHX8 can directly bind to the LHX8 DNA binding motif in the *Lin28a* promoter

We tested whether LHX8 can bind to the *Lin28a* promoter. LHX8 is an oocyte-specific LIM homeodomain transcriptional regulator that is predicted to bind DNA. A previous study showed that the LHX8 homeodomain preferentially binds a TGATTG DNA motif [22]. We identified a single TGATTG DNA motif −531 to −536 bp upstream of the putative transcriptional initiation site in the *Lin28a* gene (Fig. 4d). The *Lin28a* TGATTG motif is conserved in other mammals, including humans. We performed a chromatin immunoprecipitation (ChIP) experiment on wild-type oocytes, using our highly specific and affinity purified anti-LHX8 antibodies [13]. Anti-LHX8 antibodies preferentially immunoprecipitated the *Lin28a* promoter DNA fragment containing the TGATTG motif (Fig. 4e, f). These data further suggest that LHX8 represses *Lin28a* expression by directly binding to the *Lin28a* promoter.

### *Lin28a* deficiency partially rescues the *Lhx8*^*flx*/*flx*^*Gdf9Cre* phenotype

Our data indicates that LHX8 suppresses *Lin28a* expression. Since LIN28A is a growth-promoting factor [17, 23] preferentially expressed in oocytes, we hypothesized that *Lin28a* deficiency will rescue *Lhx8*^*flx*/*flx*^*Gdf9Cre*-induced PFA. We bred *Lin28a* and *Lhx8* floxed mice with *Gdf9Cre* to generate *Lhx8*/*Lin28a* double conditional knockouts (*Lhx8*^*flx*/*flx*^*Lin28a*^*flx*/*flx*^*Gdf9Cre*).

**Fig. 5:**
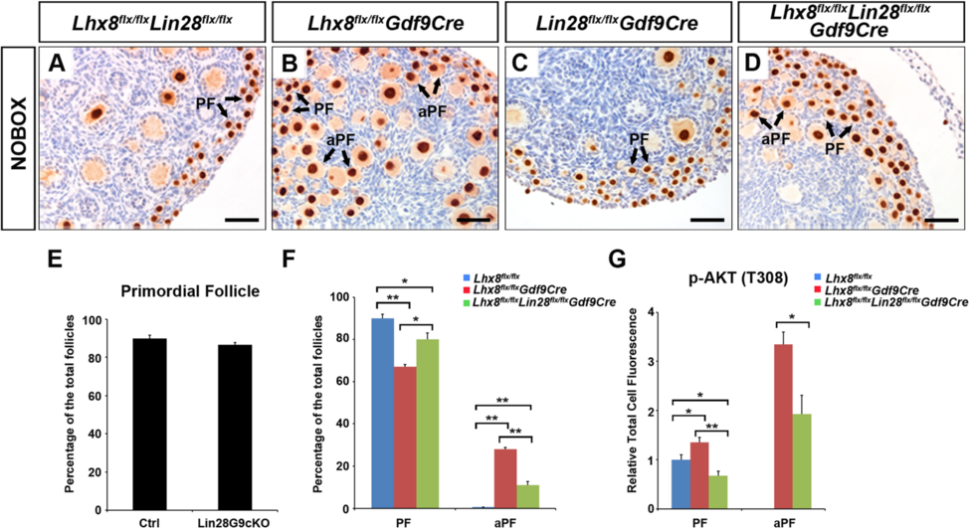
*Lin28a* deficiency rescues *Lhx8* ^*flx*/*flx*^ *Gdf9Cre*-induced PFA. **a**–**d** Representative histology of control (*Lhx8* ^*flx*/*flx*^), *Lhx8* conditional knockout (*Lhx8* ^*flx*/*flx*^ *Gdf9Cre*), *Lin28a* conditional knockout (*Lin28* ^*flx*/*flx*^ *Gdf9Cre*), and double *Lhx8*/*Lin28a* conditional knockout (*Lhx8* ^*flx*/*flx*^ *Lin28* ^*flx*/*flx*^ *Gdf9Cre*) ovaries. Double *Lhx8*/*Lin28a* conditional knockouts show a diminished number of activated primordial follicles. Anti-Nobox antibodies were used to label oocyte nuclei (dark brown). PF: primordial follicle; aPF: activated primordial follicle. **e** The *Lin28* ^*flx*/*flx*^ *Gdf9Cre* (Lin28G9cKO) mice have a similar phenotype as control (Ctrl) mice and the primordial follicle count is expressed as the percentage of total follicles. **f** Quantitation of activated primordial follicles in control, *Lhx8* conditional, and double *Lhx8*/*Lin28a* conditional knockouts. The percentage of primordial follicles (PF) and activated primordial follicles (aPF) is shown. Three pairs of ovaries from *Lhx8* conditional knockout and double *Lhx8*/*Lin28a* conditional knockouts mice were serially sectioned, and every fifth section was counted. **g** AKT phosphorylation is diminished in double *Lhx8*/*Lin28a* conditional knockouts. We quantitated by fluorescence p-AKT (T308) expression in the primordial and activated primordial follicles of the *Lhx8* ^*flx*/*flx*^ *Gdf9Cre* (G9cKO) and *Lhx8* ^*flx*/*flx*^ *Lin28a* ^*flx*/*flx*^ *Gdf9Cre* (G9dKO) ovaries at PD7. There was a significant decrease in AKT phosphorylation in *Lhx8* ^*flx*/*flx*^ *Lin28a* ^*flx*/*flx*^ *Gdf9Cre* primordial and activated primordial follicles compared to *Lhx8* ^*flx*/*flx*^ *Gdf9Cre*. Student’s *t*-test was used to calculate *P* values. *P < 0.05, **P < 0.01. Scale bars: 50 μm (A–D)

We performed ovarian morphometric analyses to determine the effects of the double knockout on PFA. No morphometric difference was observed between *Lin28a*^*flx*/*flx*^*Gdf9Cre* and control females at PD7 (Fig. 5a, c, e). As expected, we observed significantly fewer activated primordial follicles in *Lhx8*^*flx*/*flx*^*Lin28a*^*flx*/*flx*^*Gdf9Cre* compared to *Lhx8*^*flx*/*flx*^*Gdf9Cre* ovaries, but they were not completely normal compared with the control (Fig. 5a, b, d, f).

The partial rescue of *Lhx8*^*flx*/*flx*^*Gdf9Cre*-induced PFA by *Lin28a* deficiency argues that *Lin28a* is a regulator of oocyte growth. The diminished number of activated primordial follicles in *Lhx8*^*flx*/*flx*^*Lin28a*^*flx*/*flx*^*Gdf9Cre* ovaries suggests that AKT pathway activation is also diminished. We assayed the p-AKT (T308) signal in primordial and activated primordial follicles of *Lhx8*^*flx*/*flx*^*Lin28a*^*flx*/*flx*^*Gdf9Cre* and *Lhx8*^*flx*/*flx*^*Gdf9Cre* ovaries (Fig. 5g) and found its expression was significantly reduced in the *Lhx8*^*flx*/*flx*^*Lin28a*^*flx*/*flx*^*Gdf9Cre* ovary compared to the ovaries of *Lhx8*^*flx*/*flx*^*Gdf9Cre* mice.

### *Lhx8* regulates the primary to secondary follicle transition

**Fig. 6:**
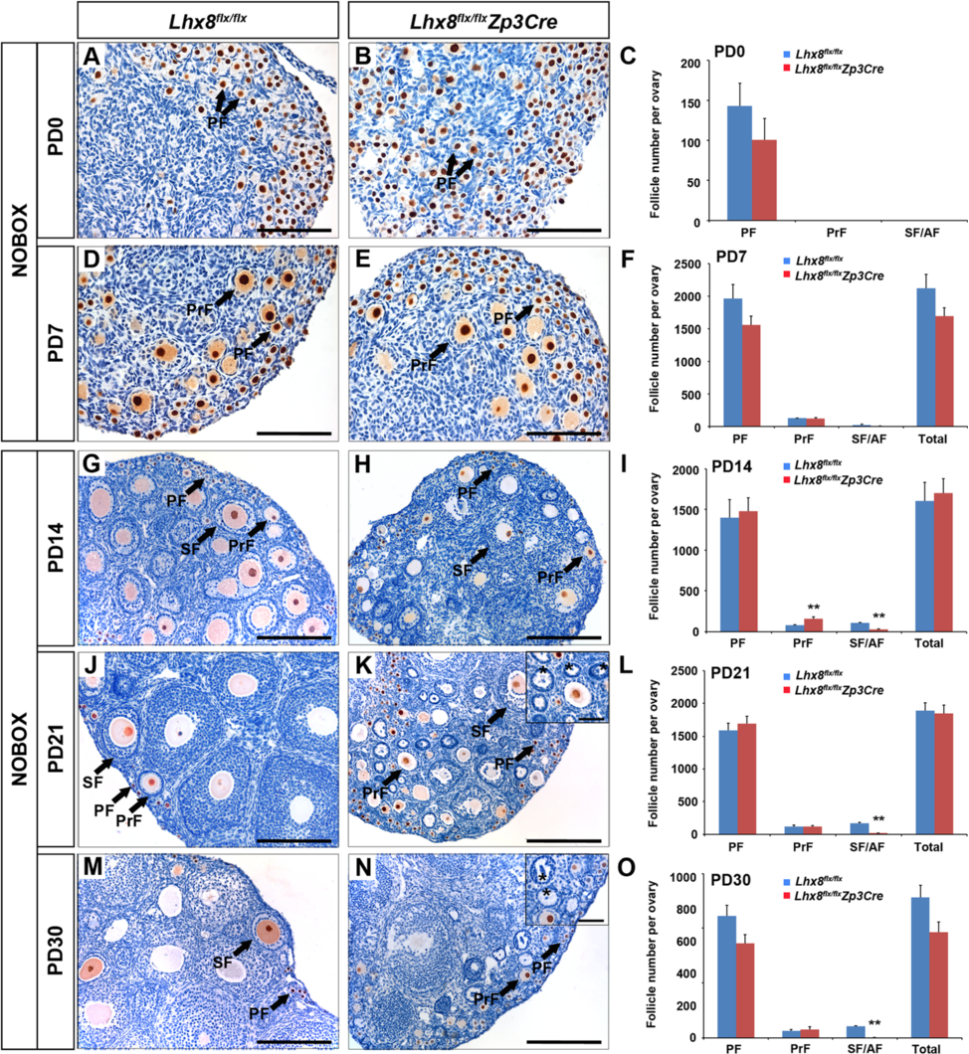
*Lhx8* inactivation in primary follicles (*Lhx8* ^*flx*/*flx*^ *Zp3Cre*) abolishes follicle growth. Histomorphological analysis was done on control (*Lhx8* ^*flx*/*flx*^) and *Lhx8* deficient ovaries (*Lhx8* ^*flx*/*flx*^ *Zp3Cre*) at various stages of postnatal ovarian development ranging from newborn (PD0) to postnatal day 30 (PD30). **a**–**f** Periodic acid–Schiff (PAS) staining and counting of different follicle types in the newborn and PD7 ovaries showed no significant differences between control and *Lhx8* deficient ovaries. **g**–**o** Anti-NOBOX antibodies were used to stain oocytes (brown immunoreactivity) in ovaries from PD14 (G and H), PD21 (J and K), and PD30 (M and N) mice. At PD14, the *Lhx8* ^*flx*/*flx*^ *Zp3Cre* ovaries showed a significantly higher number of primary follicles (PrF) and significantly diminished number of secondary/preantral (SF/AF) follicles characterized by two or more layers of granulosa cells. At PD21 and PD30, the primary follicle pool did not differ significantly between *Lhx8* ^*flx*/*flx*^ *Zp3Cre* and control ovaries; however, there was a marked decrease in the number of secondary and more advanced ovarian follicles in conditional knockouts including degenerating follicles without oocytes (marked by *asterisks* in *insets* in K and N). The primordial follicle (PF) pool remained relatively stable between PD14 and PD30, with no significant difference between *Lhx8* ^*flx*/*flx*^ *Zp3Cre* and control ovaries. **P < 0.01. Scale bars: 100 μm (A, B, D, and E); 200 μm (G, H, J, K, M, and N); 50 μm (*insets* of K and N)

Previous studies have shown that PTEN-regulated pathways are important in primordial oocyte activation, but not in primary oocytes [24]. We studied the role of *Lhx8* in primary oocytes by generating *Lhx8*^*flx*/*flx*^*Zp3Cre* mice. *Zp3Cre* is specifically expressed in primary oocytes, and *Lhx8*^*flx*/*flx*^*Zp3Cre* ovaries continue to express LHX8 in primordial, but not primary, oocytes. Morphometric analyses revealed that the *Lhx8*^*flx*/*flx*^*Zp3Cre* conditional knockout ovaries did not significantly differ from *Lhx8*^*flx*/*flx*^ (control) mice at PD0 and PD7 (Fig. 6a–f). However, at PD14, we counted 159 ± 23 primary follicles and 29 ± 7 secondary/antral follicles per ovary in the *Lhx8*^*flx*/*flx*^*Zp3Cre* mice, compared to 79 ± 6 primary follicles and 108 ± 7 secondary/antral follicles per ovary in the control mice (Fig. 6g–i). The relative increase of primary follicles at PD14 and the relative decrease of multilayer follicles in the *Lhx8*^*flx*/*flx*^*Zp3Cre* ovary indicated that the transition from primary follicles to secondary follicles was blocked, which was consistent with the observation in *Lhx8*^*flx*/*flx*^*Gdf9Cre* mice (Fig. 1f). At PD21 and PD30, we observed that many primary follicles were devoid of oocytes (Fig. 6k, n). We stained for LIN28A in PD21 ovaries and found that LIN28A was strongly expressed in *Lhx8* deficient oocytes of the *Lhx8*^*flx*/*flx*^*Zp3Cre* ovary, but no expression was detected in the empty follicles (see Additional file 6: Figure S5). However, excluding these empty primary follicles, the number of primary follicles between control and *Lhx8*^*flx*/*flx*^*Zp3Cre* ovaries was not significantly different at PD21 or PD30 (Fig. 6l, o). For secondary/antral follicles, the number sharply dropped to 17 ± 3 at PD21 and to 2 ± 1 at PD30 in *Lhx8*^*flx*/*flx*^*Zp3Cre* ovaries, compared to 170 ± 2 and 104 ± 4, respectively, in control mice. These findings imply that the growing follicle pool continued to be eliminated from the *Lhx8*^*flx*/*flx*^*Zp3Cre* mice. *Lhx8*^*flx*/*flx*^*Zp3Cre* mice were infertile (see Additional file 2: Figure S2A) and superovulation treatment of *Lhx8*^*flx*/*flx*^*Zp3Cre* mice did not produce oocytes (see Additional file 2: Figure S2B). This result was in accord with the sharp fall of secondary/antral follicles in the *Lhx8*^*flx*/*flx*^*Zp3Cre* ovary at PD21. Taken together, these data show that the folliculogenesis of *Lhx8*^*flx*/*flx*^*Zp3Cre* mice is blocked in the transition from the primary to secondary follicle stage and results in primary oocyte death and infertility. The relative stability of the primordial follicle pool from PD14 to PD30 suggests that the PFA into primary follicles was not affected by the diminution in the number of secondary and more advanced ovarian follicles in *Lhx8*^*flx*/*flx*^*Zp3Cre* ovaries.

## Discussion

Early folliculogenesis in mammals is one of the least understood frontiers in ovarian follicle biology. Primordial follicles in mice form shortly after birth and remain dormant for prolonged intervals until individual follicles resume growth via little-understood mechanisms of PFA. Several breakthrough studies have highlighted the importance of the PI3K-AKT-mTORC pathways in PFA [5–8, 25, 26]. The Kit receptor mediates the action of the PI3K-AKT-mTORC pathways [27]. Moreover, recent studies show that the Hippo pathway is also involved in PFA [28]. These ubiquitous pathways clearly play important roles in many biological processes and tissues, including oocytes. The role of oocyte-specific pathways in PFA, however, is less clear.

Our previous studies on global knockouts of *Lhx8* showed rapid loss of oocytes shortly after birth [13, 14, 29, 30]. The expression of *Lhx8* is specifically confined to oocytes and commences during the meiotic prophase in the embryonic gonad and persists in primordial, primary, and antral oocytes during both early development and adulthood. To eliminate any effects of *Lhx8* deficiency in the embryonic gonad, we utilized floxed *Lhx8* mice and the efficient *Gdf9Cre* and *Zp3Cre* mice [16] to delete conditionally *Lhx8* alleles in the oocytes of primordial and primary oocytes, respectively. Our results show that *Lhx8* represses the earliest stage of PFA, oocyte activation, and is crucial for the differentiation of primary follicles.

The massive PFA caused by depletion of *Lhx8* is somewhat reminiscent of the PI3K-Akt-mTORC1 activation caused by conditional deletion of its inhibitors, *Pten* [6] and *Tsc1* [5]. However, unlike the PI3K-AKT-mTOR related experiments, LHX8 appears to act earlier as shown by the decoupling of oocyte activation and somatic cell transformation from flat to cuboidal granulosa cells, by earlier loss of oocytes from *Lhx8*^*flx*/*flx*^*Gdf9Cre* ovaries, and by LHX8 being upstream of the KIT receptor [14]. PTEN antagonizes PI3K, and removal of PTEN activates AKT, which in turn, phosphorylates a wide range of intracellular targets, including FOXO3. FOXO3 is a specific target of the AKT pathway, and FOXO3 phosphorylation via AKT is thought to cause FOXO3 nuclear export and PFA. Our studies show that cross-talk exists between LHX8 and the PI3K-AKT-mTORC1 pathways. AKT phosphorylation is elevated in the activated oocytes of *Lhx8*^*flx*/*flx*^*Gdf9Cre* ovaries and *Lhx8*/*Pten* double knockouts, which synergistically affects FOXO3 nucleocytoplasmic localization and rpS6 activation. The FOXO3 nucleocytoplasmic shuttling may not be the first step in initiating oocyte activation, because more than 70 % of unactivated primordial follicles (<20 μm) show FOXO3 nucleocytoplasmic translocation in the double *Pten*/*Lhx8* conditionally deficient ovaries, and FOXO3 nucleocytoplasmic shuttling was mostly observed in oocytes greater than 30 μm among single *Lhx8* conditional knockouts. Moreover, the *Lhx8* conditional knockout phenotype is dominant to *Pten* as *Pten*/*Lhx8* double conditional knockouts cannot rescue *Lhx8* induced oocyte loss, and ovaries are indistinguishable from *Lhx8* single conditional knockout ovaries (see Additional file 1: Figure S1).

The expression of genes that are part of the PI3K-AKT-mTORC1 pathways was not significantly affected by conditional inactivation of LHX8 in oocytes by both quantitative PCR and RNA-seq (Fig. 2 and Additional file 5: Table S1). These results indicate that LHX8 indirectly interacts with the members of the PI3K-AKT-mTORC1 pathways to affect PFA. Genes known to affect folliculogenesis, such as *Gdf9*, *Bmp15*, and *Amh*, were downregulated (see Additional file 5: Table S1). The lack of GDF9 and BMP15 is unlikely to explain the decoupling of oocyte activation and somatic cell differentiation as *Gdf9* and *Bmp15* knockouts form primary follicles [31]. It is likely that factors other than GDF9 and BMP15 are involved in primordial oocyte-soma communication. RNA-seq identified a sixfold upregulation of *Lin28a* in *Lhx8*^*flx*/*flx*^*Gdf9Cre* ovaries. Real-time PCR confirmed the upregulation of *Lin28a* in *Lhx8*^*flx*/*flx*^*Gdf9Cre* ovaries, but *Lin28b* was not significantly changed (see Additional file 7: Figure S6A). In addition, *Lin28b* was not modified in *Lhx8*^*flx*/*flx*^*Lin28a*^*flx*/*flx*^*Gdf9Cre* ovaries either (see Additional file 7: Figure S6B). LHX8 can bind to the conserved LHX8 DNA binding motif in the *Lin28a* promoter, and *Lin28a* deficiency suppresses primordial oocyte activation observed in *Lhx8* deficient oocytes. Moreover, *Lin28a* deficiency reduced the AKT activation observed in LHX8 deficient oocytes. These experiments show that LHX8 indirect regulation of the AKT activation is mediated in part via LIN28A. Interestingly, LIN28A overexpression in myoblasts enhanced phosphorylation of AKT and mTORC1 signaling targets [21]. *Lin28a* is preferentially expressed in the gonads and germline [32]. In *Caenorhabditis elegans*, loss of *lin*-*28* results in precocious vulva differentiation and premature developmental progression [33]. In mice, *Lin28a* gain-of-function leads to a delay in mouse puberty, with increased body size [17]. The mechanism behind LIN28A somatic growth-promoting functions in mammals involves increased glucose utilization in part via an increase in insulin-PI3K-mTOR signaling [17, 21]. Besides, LIN28A is also a well-known RNA-binding protein that blocks biogenesis of many microRNAs [34–36]. Although previous studies have shown that loss of LIN28A function in oocytes does not affect fertility or oogenesis [37], our results suggest that LIN28A overexpression, under the control of LHX8, regulates PFA.

We examined expression of *let*-*7* microRNAs in *Lhx8*^*flx*/*flx*^*Gdf9Cre* and *Lhx8*^*flx*/*flx*^*Lin28a*^*flx*/*flx*^*Gdf9Cre* oocytes. *Let*-*7* transcript levels were not changed in either mouse model (see Additional file 8: Figure S7). These results are consistent with a previous report showing that *Lin28a* and *Lin28b* do not regulate *let*-*7a* in oocytes [37]. Given that *Lin28a* deficiency reduced the AKT activation observed in LHX8 deficient oocytes, we consider that LIN28A regulates AKT activation through a way independent of *let*-*7*. LIN28A binds many RNA species, and further investigations are needed to identify LIN28A’s mechanisms of action in oocytes [38, 39].

Unlike PTEN-mediated pathways, whose actions are stage-specific and confined to PFA [24], *Lhx8* deletion in primary oocytes disrupts follicle growth beyond primary oocytes and results in infertility. The initial rise in primary follicles circa PD14 is likely due to the onset of the *Zp3Cre*-mediated inactivation of *Lhx8* that blocks the transition from primary to more advanced follicles. At PD21 and PD30, oocyte death in primary follicles is evident. Interestingly, we did not observe a significant decline in primordial follicles from PD0 to PD30, which argues against the notion that secondary and more advanced follicles inhibit PFA.

## Conclusions

Our study shows that *Lhx8* plays a critical role in primordial oocyte activation and somatic differentiation and proliferation. *Lhx8*-regulated pathways are dominant over PTEN pathways, are upstream of KIT, and unlike PTEN pathways, are required for later stages of oogenesis. Multiple pathways, both ubiquitous (PI3K-AKT and Hippo) and oocyte-specific (LHX8), play important roles in oocyte activation and survival. These models will allow further molecular dissection of oocyte-specific activation and signaling that allows the somatic component to differentiate from pre-granulosa cells to cuboidal and multi-layered follicles.

## Methods

### Mice

The conditional knockout of *Lhx8* was established by mating *Lhx8*^*flx*/*flx*^ (C57BL/6 J-129/Ola) mice and *Gdf9Cre* (C57-B6/SJL) or *Zp3Cre* mice (C57-BL/6 J) [15, 16, 40], to create *Lhx8*^*flx*/*flx*^*Gdf9Cre* mice. The *Pten*^*flx*^ mice were obtained from the Jackson Laboratory (Bar Harbor, MN) [41]. Homozygous floxed B6;129-*Lin28a*^*tm1Egm*^ mice were purchased from Jackson Laboratory [42].

### Histology, immunohistochemistry, and immunofluorescence analysis

Ovaries were fixed in 10 % buffered formalin or 4 % paraformaldehyde at room temperature for less than 24 h, processed, embedded in paraffin, serially sectioned (5 μm), and stained by periodic acid–Schiff (PAS) reagent (#26052-05, Electron Microscopy Sciences, Hatfield, PA) and Mayer’s hematoxylin counterstain for histological analysis. The immunohistochemistry was studied as previously described [13]. Affinity purified anti-LHX8 antibodies were generated as previously described [13]. Antibodies against p-rpS6 (S235/236, #2211) were purchased from Cell Signaling Technology (Danvers, MA). Immunofluorescence was performed as previously described [43]. Antibodies against FOXO3, LIN28A, p-AKT (S473), and p-AKT (T308) were purchased from Cell Signaling Technology (Danvers, MA) and Millipore (Temecula, CA). The immunofluorescence intensity of p-AKT (T308) was measured using ImageJ software [44, 45]. Corrected total cell fluorescence (CTCF) was used for statistical analysis [44, 45]: CTCF = Integrated density – (Area of selected cell × Mean fluorescence of background readings).

### RNA isolation, reverse transcription, RT-qPCR, and RNA-seq analysis

RNA from PD7 ovaries was extracted using the RNeasy Mini Kit (Qiagen, Valencia, CA) and RNA from PD7 oocytes was extracted by the ARCTURUS PicoPure RNA Isolation Kit (Life Technologies, Grand Island, NY), then further subjected to DNase digestion with the RNase-Free DNase Set (Qiagen) and RNA cleanup. RT-qPCR was performed with the CFX96 Real-Time PCR Detection System (Bio-Rad, Hercules, CA). Each experiment was repeated with a minimum of three independent samples, and results were normalized to *Gapdh*. The relative amount of transcripts was calculated by the ΔΔCT method. Significance was calculated using Student’s *t*-test with Excel (Microsoft).

For RNA-seq, ten pairs of control and *Lhx8* conditionally deficient PD7 ovaries were used to extract RNA; a TruSeq RNA sample prep kit (Illumina, San Diego, CA) was used to generate a cDNA library. Approximately 180 million reads were generated per sample (control and cKO cDNA) on Hi-Seq 2000 (Illumina, San Diego). The RNA-seq reads were analyzed using TopHat [46], Cufflinks [47], and Cuffdiff [48]. The raw RNA-seq data from this study have been submitted to the Sequence Read Archive of the National Center for Biotechnology Information with accession number SRP047051.

### Oocyte isolation and Western blots

Oocytes were isolated according to the method described by Reddy et al. [26]. In total, 400 oocytes from control or *Lhx8*^*flx*/*flx*^*Gdf9Cre* ovaries were isolated and used for Western blots. Primary antibodies of LIN28A (#8641), p-AKT (S473, #9271), p-AKT (T308, #2965), AKT (#9272), and histone H3 (#4499) were purchased from Cell Signaling.

### Oocyte ChIP and ChIP-qPCR

Oocyte ChIP was performed following a micro-chromatin immunoprecipitation protocol [49] with some modification. Oocytes were isolated from five PD5 wild-type mice and fixed with 37 % formalin in a 1 % final concentration for 10 min. Chromatin fragmentation was conducted using 1 U micrococcal nuclease for 15 min at 37 °C, following the manufacturer’s manual (Pierce Chromatin Prep Module, #26158, Pierce Biotechnology, Rockford, IL). Chromatin was dissolved in 100 μl nuclear extraction buffer, and affinity purified LHX8 antibody [13] or normal guinea pig IgG (sc-2711) were used for immunoprecipitation. Immunoprecipitated DNA was subjected to real-time PCR analysis using the primers corresponding to the *Lin28a* promoter region that contains the putative LHX8 DNA binding element [22]. Primer set 1 forward: 5′-gagggggagggctcatccatttct-3′, reverse: 5′-ttccagagagttgggggagggagg-3′ (−584 to −485); and primer set 2 forward: 5′-gctcatccatttctcccttgacagg-3′, reverse: 5′-acacacacacacacacacaccacc-3′ (−574 to −434).

### Statistics

Student’s *t*-test was performed for comparative studies. *P* < 0.05 was considered statistically significant.

## Additional files

Additional file 1: Figure S1. AKT pathway activation does not rescue oocyte death in *Lhx8* conditionally deficient ovaries. Ovaries were harvested at PD35 and examined for the presence of follicles in control (*Lhx8* ^*flx*/*flx*^, **A**), *Pten* conditional knockout (*Pten* ^*flx*/*flx*^ *Gdf9Cre*, **B**), *Lhx8* conditional knockout (*Lhx8* ^*flx*/*flx*^ *Gdf9Cre*, **C**), and double *Lhx8*/*Pten* conditional knockout (*Lhx8* ^*flx*/*flx*^ *Pten* ^*flx*/*flx*^ *Gdf9Cre*, **D**) ovaries. *Lhx8* ^*flx*/*flx*^ *Gdf9Cre* mice lose oocytes rapidly, so that by PD35 few remain. This is distinctly different from *Pten* ^*flx*/*flx*^ *Gdf9Cre* mice, whose activated oocytes persist beyond 5 weeks [6], as PTEN deletion activates the AKT pathway by increased AKT phosphorylation, which results in increased cell survival. The double knockout histology was not significantly different from *Lhx8* conditional knockout and indicates that the *Lhx8* pathway is dominant to the *Pten* pathway in controlling oocyte survival. Scale bars: 100 μm. Additional file 2: Figure S2. Fertility test of *Lhx8* ^*flx*/*flx*^ *Zp3Cre* mice. **A** *Lhx8* ^*flx*/*flx*^ *Gdf9Cre* (G9cKO) mice and *Lhx8* ^*flx*/*flx*^ *Zp3Cre* (Zp3cKO) mice were infertile. **B** To test the ovulation ability of the *Lhx8* ^*flx*/*flx*^ *Zp3Cre* mice, 3-week-old mice were intraperitoneally injected with 5 IU pregnant mare serum gonadotropin (PMSG). After 48 hours, 5 IU human chorionic gonadotropin (HCG) was injected. Then 24 hours later, the ovary and oviduct were collected together to flush oocytes. Additional file 3: Figure S3. AKT expression in *Lhx8* conditional knockout ovaries. **A**–**F** Anti-p-AKT (S473) antibodies show p-AKT(S473) expression mainly in the cytoplasm of primary oocytes in the control (*Lhx8* ^*flx*/*flx*^) and *Lhx8* conditional knockout (*Lhx8* ^*flx*/*flx*^ *Gdf9Cre*) ovaries, but were not significantly detected in activated primordial follicles (aPF, *arrows* in **F**'). **G**–**L** p-AKT (T308) was firmly located in the primary follicles (PrF, *arrows*) or the transitional follicles (PF/PrF, *arrow* in **I**'), both in control (*Lhx8* ^*flx*/*flx*^) and *Lhx8* conditional knockout (*Lhx8* ^*flx*/*flx*^ *Gdf9Cre*) ovaries. Unlike p-AKT (S473), activated primordial follicles in *Lhx8* conditional knockouts express p-AKT (T308) (aPF, *arrows* in **L**'). The *boxed areas* in panels C, F , I, and L are shown magnified in C', F', I', and L'. Scale bars: 50 μm (A–L); 20 μm (C', F', I' and L'). Additional file 4: Figure S4.
*Lhx8 and Pten* conditional knockouts show synergism in p-rpS6 activation. Ovaries from control (*Lhx8* ^*flx*/*flx*^, **A** and **A**'), *Lhx8* conditional knockout (*Lhx8* ^*flx*/*flx*^ *Gdf9Cre*, **B** and **B**'), *Pten* conditional knockout (*Pten* ^*flx*/*flx*^ *Gdf9Cre*, **C** and **C**'), and double conditional knockout (*Lhx8* ^*flx*/*flx*^ *Pten* ^*flx*/*flx*^ *Gdf9Cre*, **D** and **D**') mice, were subjected to immunohistochemistry using anti-p-rpS6 antibodies. In the control ovaries and single conditional knockouts, p-rpS6 is expressed primarily in primary or secondary follicles, but not in the vast majority of primordial follicles (PF) or activated primordial follicles (aPF), except for occasional cells (*arrowhead* in B'). However, in the double conditional knockout mice, the majority of activated primordial follicles are p-rpS6 positive (*arrows*). Scale bars: 100 μm (A–D); 20 μm (A'–D'). Additional file 5: Table S1. RNA-seq data derived from PD7 control (*Lhx8* ^*flx*/*flx*^, Ctrl) and *Lhx8* ^*flx*/*flx*^ *Gdf9Cre* (G9cKO) ovaries. FPKM: Fragments per kilobase of transcript per million mapped reads. The *P* value is calculated by Cuffdiff software; a *P* value of less than 0.05 is taken as significant. Additional file 6: Figure S5. LIN28A expression in PD21 *Lhx8* ^*flx*/*flx*^ and *Lhx8* ^*flx*/*flx*^ *Zp3Cre* ovaries. In PD21 *Lhx8* ^*flx*/*flx*^ control ovaries, LIN28A has weak expression in big follicles (*arrows*) (**A**–**D**), but it was still overexpressed in LHX8 depletion oocytes of *Lhx8* ^*flx*/*flx*^ *Zp3Cre* mice (*arrows*) (**E**–**H**). However, follicles containing dead oocytes had no LIN28A expression (*asterisk*). Scale bars: 50 μm. Additional file 7: Figure S6.
*Lin28a* and *Lin28b* expression in PD7 control (*Lhx8* ^*flx*/*flx*^ and *Lhx8* ^*flx*/*flx*^ *Lin28a* ^*flx*/*flx*^), *Lhx8* ^*flx*/*flx*^ *Gdf9Cre*, and *Lhx8* ^*flx*/*flx*^ *Lin28a* ^*flx*/*flx*^ *Gdf9Cre* ovaries. **A** *Lin28a* and *Lin28b* transcription was examined by real-time PCR in PD7 control (*Lhx8* ^*flx*/*flx*^) and *Lhx8* ^*flx*/*flx*^ *Gdf9Cre* mouse ovaries. **B** *Lin28a* and *Lin28b* transcription was examined by real-time PCR in PD7 control (*Lhx8* ^*flx*/*flx*^ *Lin28a* ^*flx*/*flx*^) and *Lhx8* ^*flx*/*flx*^ *Lin28a* ^*flx*/*flx*^ *Gdf9Cre* mouse ovaries. Data were normalized to *Gapdh* expression and are given as the mean relative quantity (compared with control), with error bars representing the standard error of the mean. Student’s *t*-test was used to calculate *P* values. ** *P*< 0.01. Additional file 8: Figure S7. Expression of *let*-*7* in *Lhx8* ^*flx*/*flx*^, *Lhx8* ^*flx*/*flx*^ *Gdf9Cre*, *Lhx8* ^*flx*/*flx*^ *Lin28a* ^*flx*/*flx*^ *Gdf9Cre*, and *Lhx8* ^*flx*/*flx*^ *Lin28a* ^*flx*/*flx*^ *Gdf9Cre* oocytes. Total RNA from PD7 oocytes was extracted using Trizol. The NCode VILO miRNA cDNA Synthesis Kit (Life Technologies, Grand Island, NY) was used for the reverse transcription of all the small RNAs. Real-time PCR showed that *let*-*7* was not changed in *Lhx8* ^*flx*/*flx*^ *Gdf9Cre* or *Lhx8* ^*flx*/*flx*^ *Lin28a* ^*flx*/*flx*^ *Gdf9Cre* oocytes. Data were normalized to 5s rRNA expression and are given as the mean relative quantity (compared with control), with error bars representing the standard error of the mean.

## Abbreviations

bp: base pair
ChIP: Chromatin immunoprecipitation
IgG: Immunoglobulin G
PAS: Periodic acid–Schiff
PCR: Polymerase chain reaction
PD: postnatal day
PFA: Primordial follicle activation
qPCR: Quantitative polymerase chain reaction
RNA-seq: RNA sequencing
RT-qPCR: real-time quantitative polymerase chain reaction
