# Supplementary figures and images for: Lhx8 regulates primordial follicle activation and postnatal folliculogenesis
Source: BMC Biol. 2015 Jun 16;13:39. doi: 10.1186/s12915-015-0151-3 (PMC4487509; doi:10.1186/s12915-015-0151-3)

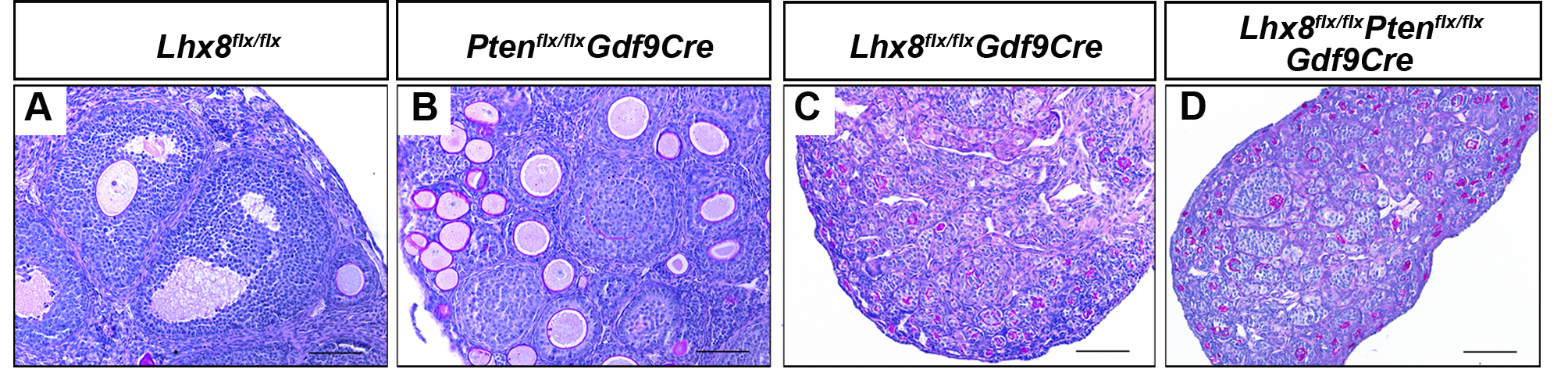

Supplement: Additional file 1: Figure S1. — AKT pathway activation does not rescue oocyte death in Lhx8 conditionally deficient ovaries. Ovaries were harvested at PD35 and examined for the presence of follicles in control (Lhx8 flx/flx, A), Pten conditional knockout (Pten flx/flx Gdf9Cre, B), Lhx8 conditional knockout (Lhx8 flx/flx Gdf9Cre, C), and double Lhx8/Pten conditional knockout (Lhx8 flx/flx Pten flx/flx Gdf9Cre, D) ovaries. Lhx8 flx/flx Gdf9Cre mice lose oocytes rapidly, so that by PD35 few remain. This is distinctly different from Pten flx/flx Gdf9Cre mice, whose activated oocytes persist beyond 5 weeks [6], as PTEN deletion activates the AKT pathway by increased AKT phosphorylation, which results in increased cell survival. The double knockout histology was not significantly different from Lhx8 conditional knockout and indicates that the Lhx8 pathway is dominant to the Pten pathway in controlling oocyte survival. Scale bars: 100 μm. [file 12915_2015_151_MOESM1_ESM.tiff]

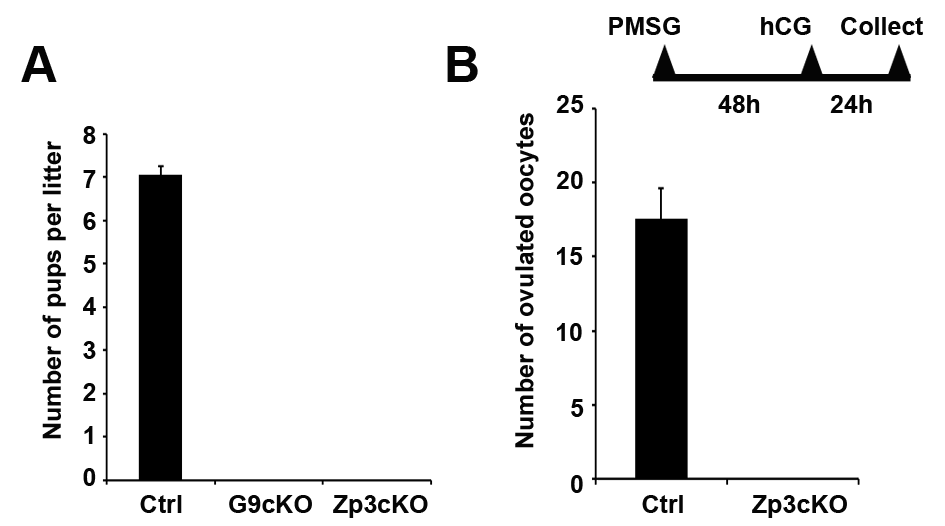

Supplement: Additional file 2: Figure S2. — Fertility test of Lhx8 flx/flx Zp3Cre mice. A Lhx8 flx/flx Gdf9Cre (G9cKO) mice and Lhx8 flx/flx Zp3Cre (Zp3cKO) mice were infertile. B To test the ovulation ability of the Lhx8 flx/flx Zp3Cre mice, 3-week-old mice were intraperitoneally injected with 5 IU pregnant mare serum gonadotropin (PMSG). After 48 hours, 5 IU human chorionic gonadotropin (HCG) was injected. Then 24 hours later, the ovary and oviduct were collected together to flush oocytes. [file 12915_2015_151_MOESM2_ESM.tiff]

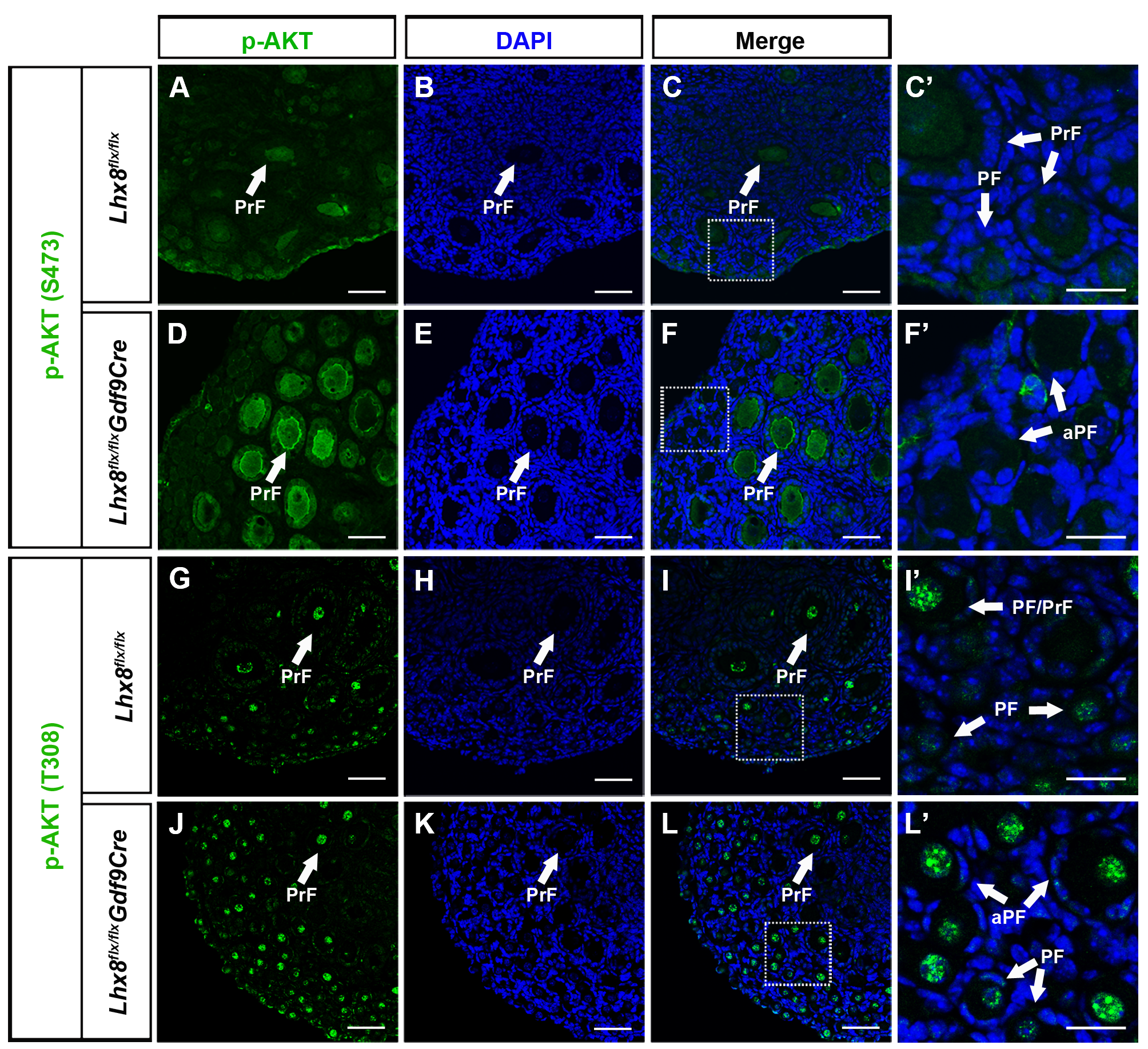

Supplement: Additional file 3: Figure S3. — AKT expression in Lhx8 conditional knockout ovaries. A–F Anti-p-AKT (S473) antibodies show p-AKT(S473) expression mainly in the cytoplasm of primary oocytes in the control (Lhx8 flx/flx) and Lhx8 conditional knockout (Lhx8 flx/flx Gdf9Cre) ovaries, but were not significantly detected in activated primordial follicles (aPF, arrows in F'). G–L p-AKT (T308) was firmly located in the primary follicles (PrF, arrows) or the transitional follicles (PF/PrF, arrow in I'), both in control (Lhx8 flx/flx) and Lhx8 conditional knockout (Lhx8 flx/flx Gdf9Cre) ovaries. Unlike p-AKT (S473), activated primordial follicles in Lhx8 conditional knockouts express p-AKT (T308) (aPF, arrows in L'). The boxed areas in panels C, F , I, and L are shown magnified in C', F', I', and L'. Scale bars: 50 μm (A–L); 20 μm (C', F', I' and L'). [file 12915_2015_151_MOESM3_ESM.tiff]

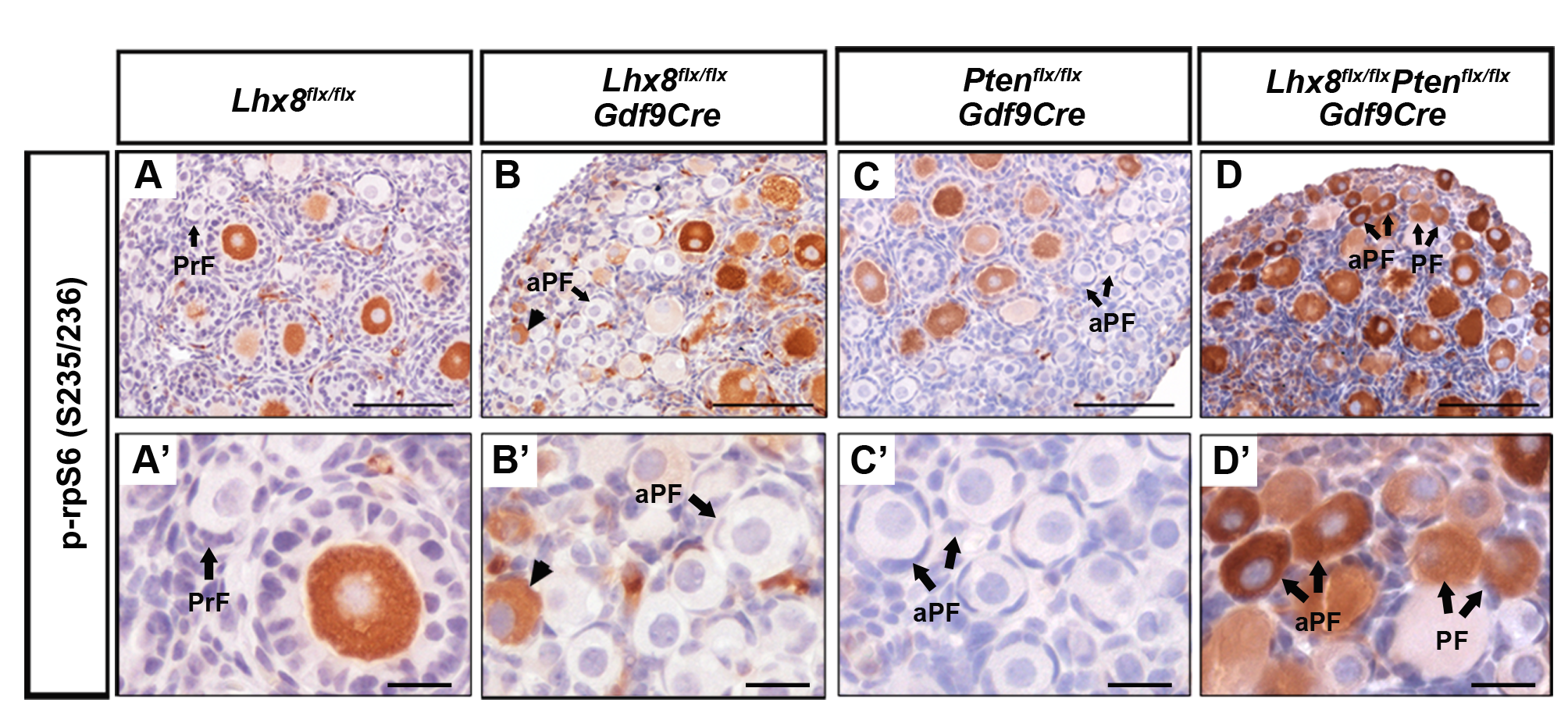

Supplement: Additional file 4: Figure S4. — Lhx8 and Pten conditional knockouts show synergism in p-rpS6 activation. Ovaries from control (Lhx8 flx/flx, A and A'), Lhx8 conditional knockout (Lhx8 flx/flx Gdf9Cre, B and B'), Pten conditional knockout (Pten flx/flx Gdf9Cre, C and C'), and double conditional knockout (Lhx8 flx/flx Pten flx/flx Gdf9Cre, D and D') mice, were subjected to immunohistochemistry using anti-p-rpS6 antibodies. In the control ovaries and single conditional knockouts, p-rpS6 is expressed primarily in primary or secondary follicles, but not in the vast majority of primordial follicles (PF) or activated primordial follicles (aPF), except for occasional cells (arrowhead in B'). However, in the double conditional knockout mice, the majority of activated primordial follicles are p-rpS6 positive (arrows). Scale bars: 100 μm (A–D); 20 μm (A'–D'). [file 12915_2015_151_MOESM4_ESM.tiff]

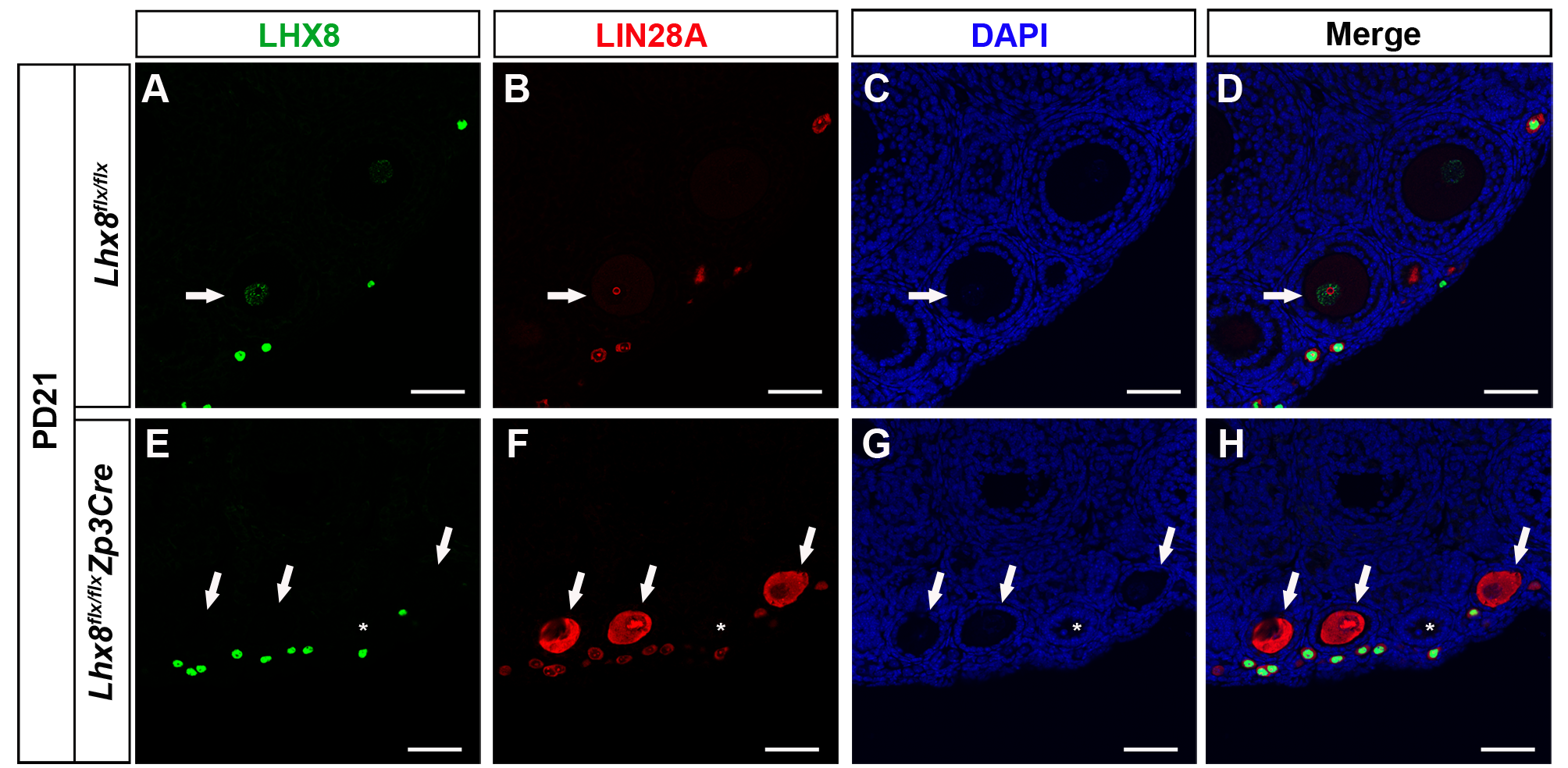

Supplement: Additional file 6: Figure S5. — LIN28A expression in PD21 Lhx8 flx/flx and Lhx8 flx/flx Zp3Cre ovaries. In PD21 Lhx8 flx/flx control ovaries, LIN28A has weak expression in big follicles (arrows) (A–D), but it was still overexpressed in LHX8 depletion oocytes of Lhx8 flx/flx Zp3Cre mice (arrows) (E–H). However, follicles containing dead oocytes had no LIN28A expression (asterisk). Scale bars: 50 μm. [file 12915_2015_151_MOESM6_ESM.tiff]

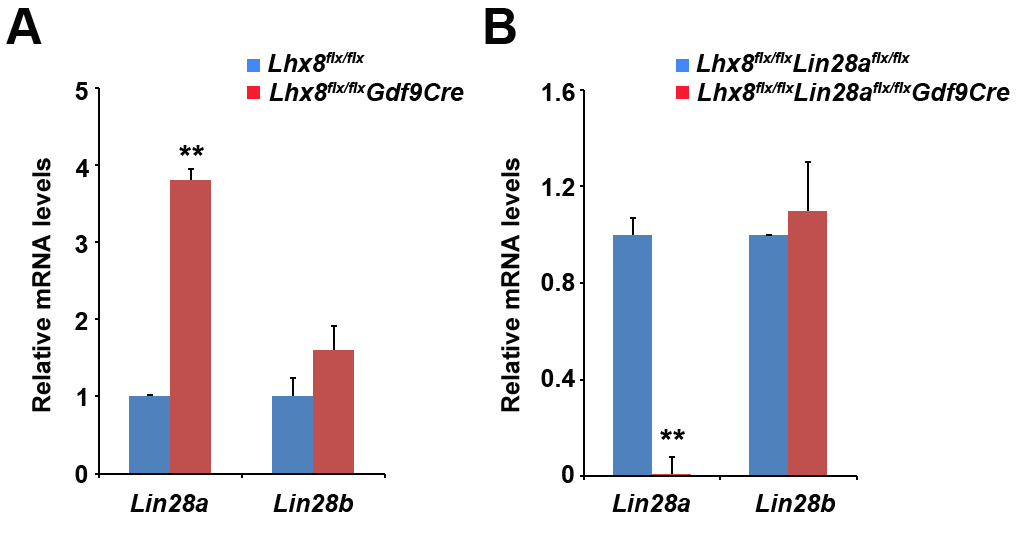

Supplement: Additional file 7: Figure S6. — Lin28a and Lin28b expression in PD7 control (Lhx8 flx/flx and Lhx8 flx/flx Lin28a flx/flx), Lhx8 flx/flx Gdf9Cre, and Lhx8 flx/flx Lin28a flx/flx Gdf9Cre ovaries. A Lin28a and Lin28b transcription was examined by real-time PCR in PD7 control (Lhx8 flx/flx) and Lhx8 flx/flx Gdf9Cre mouse ovaries. B Lin28a and Lin28b transcription was examined by real-time PCR in PD7 control (Lhx8 flx/flx Lin28a flx/flx) and Lhx8 flx/flx Lin28a flx/flx Gdf9Cre mouse ovaries. Data were normalized to Gapdh expression and are given as the mean relative quantity (compared with control), with error bars representing the standard error of the mean. Student’s t-test was used to calculate P values. ** P< 0.01. [file 12915_2015_151_MOESM7_ESM.tiff]

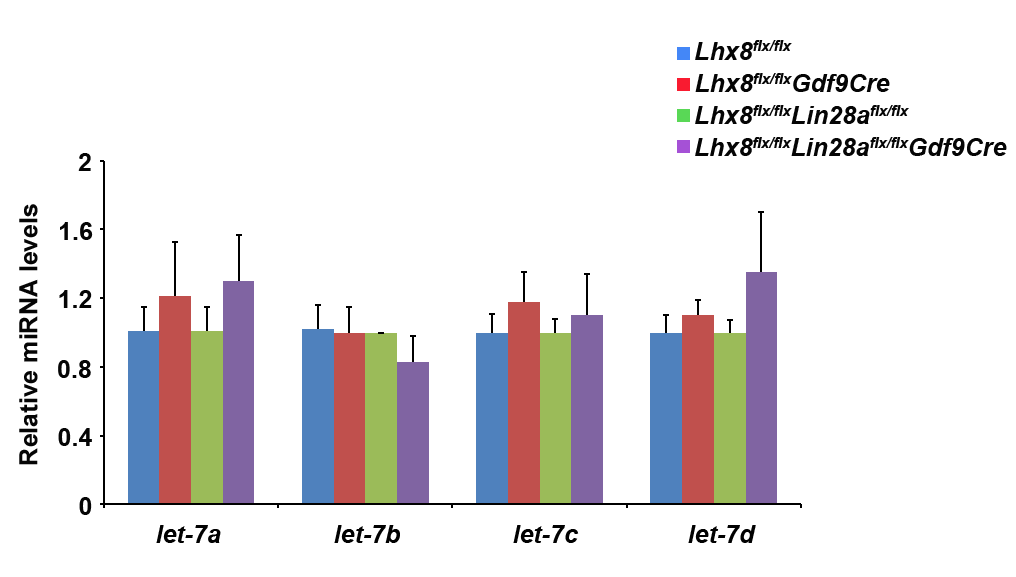

Supplement: Additional file 8: Figure S7. — Expression of let-7 in Lhx8 flx/flx, Lhx8 flx/flx Gdf9Cre, Lhx8 flx/flx Lin28a flx/flx Gdf9Cre, and Lhx8 flx/flx Lin28a flx/flx Gdf9Cre oocytes. Total RNA from PD7 oocytes was extracted using Trizol. The NCode VILO miRNA cDNA Synthesis Kit (Life Technologies, Grand Island, NY) was used for the reverse transcription of all the small RNAs. Real-time PCR showed that let-7 was not changed in Lhx8 flx/flx Gdf9Cre or Lhx8 flx/flx Lin28a flx/flx Gdf9Cre oocytes. Data were normalized to 5s rRNA expression and are given as the mean relative quantity (compared with control), with error bars representing the standard error of the mean. [file 12915_2015_151_MOESM8_ESM.tiff]
